# The Effect of Perceptual Learning on Face Recognition in Individuals with Central Vision Loss

**DOI:** 10.1167/iovs.61.8.2

**Published:** 2020-07-01

**Authors:** Elizabeth M. Haris, Paul V. McGraw, Ben S. Webb, Susana T. L. Chung, Andrew T. Astle

**Affiliations:** 1 Visual Neuroscience Group, School of Psychology, The University of Nottingham, Nottingham, United Kingdom; 2 School of Optometry, University of California, Berkeley, Berkeley, California, United States

**Keywords:** age-related macular degeneration, central vision loss, perceptual learning, face recognition, ARMD

## Abstract

**Purpose:**

To examine whether perceptual learning can improve face discrimination and recognition in older adults with central vision loss.

**Methods:**

Ten participants with age-related macular degeneration (ARMD) received 5 days of training on a *face discrimination* task (mean age, 78 ± 10 years). We measured the magnitude of improvements (i.e., a reduction in threshold size at which faces were able to be discriminated) and whether they generalized to an untrained *face recognition* task. Measurements of visual acuity, fixation stability, and preferred retinal locus were taken before and after training to contextualize learning-related effects. The performance of the ARMD training group was compared to nine untrained age-matched controls (8 = ARMD, 1 = juvenile macular degeneration; mean age, 77 ± 10 years).

**Results:**

Perceptual learning on the face discrimination task reduced the threshold size for face discrimination performance in the trained group, with a mean change (SD) of –32.7% (+15.9%). The threshold for performance on the face recognition task was also reduced, with a mean change (SD) of –22.4% (+2.31%). These changes were independent of changes in visual acuity, fixation stability, or preferred retinal locus. Untrained participants showed no statistically significant reduction in threshold size for face discrimination, with a mean change (SD) of –8.3% (+10.1%), or face recognition, with a mean change (SD) of +2.36% (–5.12%).

**Conclusions:**

This study shows that face discrimination and recognition can be reliably improved in ARMD using perceptual learning. The benefits point to considerable perceptual plasticity in higher-level cortical areas involved in face-processing. This novel finding highlights that a key visual difficulty in those suffering from ARMD is readily amenable to rehabilitation.

Our ability to recognize faces is a fundamental skill underpinning human social interaction.^1^^,^^2^ Although many people with normal visual function take face recognition for granted, those who suffer from central vision loss find it an extremely challenging task that limits social interactions and can lead to social isolation.^3^^–^^5^

Central vision loss can be caused by many factors and is commonly associated with degeneration of cones in the macular region of the retina.^6^ When the macular region becomes damaged, the ability to see fine spatial detail is lost, and tasks such as reading and face recognition are severely compromised.^3^ The most prevalent hereditary form of macular degeneration is Stargardt's disease,^7^ whereas the leading cause of vision loss in individuals over 50 years of age in the developed world is age-related macular degeneration (ARMD).^8^

The late stages of ARMD are characterized by decreased visual acuity, poor contrast sensitivity, and blind regions of the central field that eventually affect both eyes.^9^^–^^11^ As a result, individuals with advanced ARMD often learn to rely on their peripheral vision and adopt a relatively unaffected region of the retina to view objects—a preferred retinal locus (PRL).^12^ However, the PRL remains limited by these factors and by poor fixation stability,^13^ resulting in an altered pattern of familiar face recognition that shows a higher dependency on external facial features (e.g., chin, hair, face outline), as opposed to a reliance on the internal features of a familiar face (e.g., eyes, nose, mouth) that is characteristic of those with typical vision.^14^ Although it is still unclear what governs the choice of a PRL, when compared to central vision it is evident that peripheral vision is also severely affected by visual crowding and shows marked reductions in thresholds for contrast sensitivity and most other forms of spatial acuity that affect several functions, including face recognition.^15^^,^^16^

Relatively few studies have explored face recognition in patients with ARMD. Bullimore and colleagues^4^ tested face recognition in early-stage ARMD individuals and age-matched controls using a face recognition task. They found that the distance at which a face was readily recognized in individuals with central vision loss decreased by 90% relative to healthy older participants. The more advanced the ARMD, the greater the difficulty an individual experienced with identity recognition. Relative to age-matched controls, Barnes and colleagues^17^ found face discrimination to be poorer and slower in individuals with ARMD and that both contrast sensitivity and visual acuity influenced face recognition. Fixation patterns are also altered in those with ARMD when they inspect faces, as there is a marked bias toward external facial features, such as hairline and outline of face.^14^^,^^18^ Clearly, facial analysis is altered when more peripheral areas of the retina are used; however, there is a growing body of evidence suggesting that peripheral visual function can be improved using perceptual learning, indicating that the behavioral constraints limiting performance in ARMD can be modified.^11^^,^^19^^–^^21^

Perceptual learning describes a set of neural processes that allow a sensory system to improve its ability to extract information about the physical environment^22^^,^^23^ and permanently modifies neural function through experience-dependent processes.^24^^,^^25^ Improvements on sensory tasks occur as a result of repeated practice, often using near-threshold stimuli, and can produce long-lasting changes in visual detection and discrimination.^26^^–^^28^ Perceptual learning can be tightly coupled to the trained visual attributes (e.g., orientation), task (e.g., detection), and retinal location.^29^^–^^32^ Overcoming this specificity is a particular challenge for the translation of perceptual learning to clinical settings.^33^

In some visually challenged populations, trained improvements to visual performance have been found to generalize to a larger range of stimuli and tasks.^34^^,^^35^ Perceptual improvements following training in people with amblyopia have been shown to generalize to untrained contrasts,^27^^,^^36^ spatial frequencies,^34^ and even to other tasks, such as visual acuity.^37^ In peripheral vision,^37^ increases in visual span for untrained retinal loci and improvements in reading speed have been demonstrated when individuals were trained only on a letter identification task.^38^ Perceptual learning has been demonstrated using face stimuli in subjects with acquired prosopagnosia, with perceptual improvements generalizing to untrained expressions, views, and faces.^39^ Considering that the adult visual system remains plastic throughout life^25^ and learned improvements in visual performance have been found to generalize, perceptual learning is gaining traction as an effective therapeutic approach that can enhance function in visually impaired populations.^40^^–^^43^

The present study investigates the effects of perceptual learning on a *face discrimination* task in individuals with central vision loss and quantifies the generalization of learning effects using a separate famous-face *recognition* task. We also measured visual acuity, fixation stability, and PRL change to partition their contribution to face learning. To minimize the influence of age and performance effects that have previously been documented (e.g., more face identification errors in older adults and individuals with ARMD^17^, poorer fixation stability in ARMD populations^14^), a control group of age-matched older individuals with central vision loss and a stable PRL were used.

## Materials and Methods

### Experimental Design

**Figure 1.**
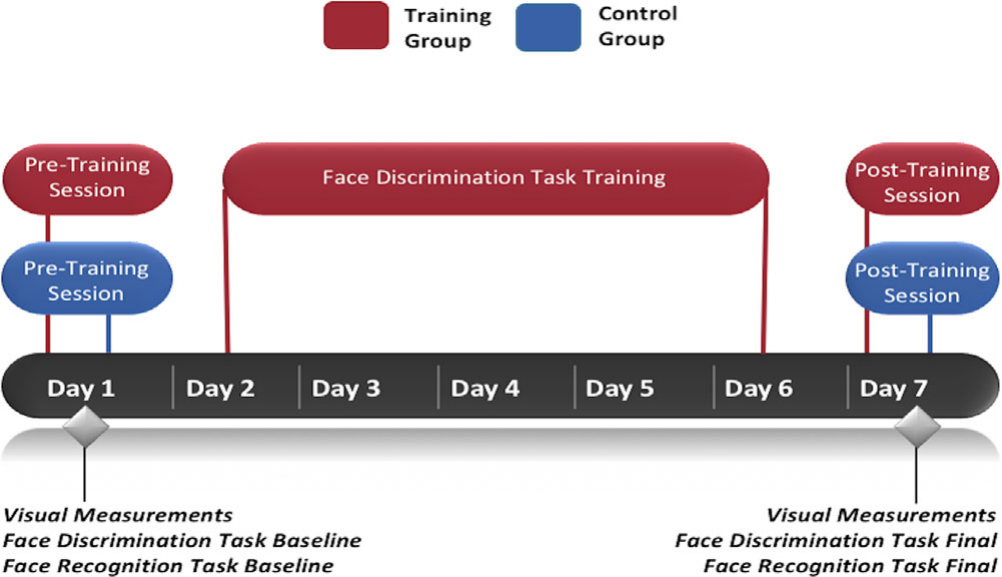
Experimental design including pre- and post-test measurements and face discrimination task training (days 2–6).

Monocular visual acuity measurements were taken pre-training to identify the eye with better acuity and were followed by measures of fixation stability and PRL estimation in that eye. Following the initial visual assessment, face discrimination and recognition were measured in a random order. After training, the order of testing was randomized (Fig. 1).

### Participants

Participants were recruited via patient databases at The University of Nottingham. Prior to taking part, the Mini-Mental State Examination was administered (cutoff ≥ 24^44^). A total of 19 participants took part: 10 trained (mean age, 78 years; SD, 10 years; age range, 62–90 years; 7 males and 3 females) and 9 control (mean age, 77 years; SD, 10 years; age range, 59–88; 3 males and 6 females). Of the 19 participants, 18 were diagnosed with ARMD and one with juvenile macular degeneration. All participants except one (NT) suffered from central vision loss in both eyes and all but one (SA) were free from injections of anti-vascular endothelial growth factor for at least 3 months prior to testing. None had existing visual comorbidities. See Table 1 for further details. The study adhered to the tenets of the Declaration of Helsinki, and informed consent was obtained prior to participation. The study was approved by the ethics committee of the School of Psychology at The University of Nottingham and by the National Health Service Research Ethics Service.

**Table 1.** Visual Characteristics and Training Details for Study Participants (*N* = 19)

						Refractive Error (Prescription) (°)		Visual Acuity (logMAR)			
Group	Participant	Age (y)	Gender	Diagnosis	Years Since Onset	Right	Left	Right	Left	Fixation Stability (BCEA 63%) (°^2^)	Trained/Tested Eye^*^
Trained	RT	82	Male	ARMD	5	+3.25/–1.75 × 95	+2.25/–1.00 × 80	0.80	1.04	9.8	Right
Trained	LJ	88	Male	ARMD	5	+5.00/–3.50 × 90	+4.50 DS	0.38	HM	0.5	Right
Trained	MV	62	Male	ARMD	16	+2.75/–0.75 × 160	pl/–1.00 × 160	0.92	0.92	6.0	Left
Trained	WB	63	Male	ARMD	20	+1.75/–3.75 × 10	+2.00/–3.50 × 5	0.88	0.66	12.9	Left
Trained	SC	79	Female	ARMD	3	pl/–1.25 × 95	+0.50/–1.75 × 80	0.56	0.44	0.2	Left
Trained	AS-1	90	Male	ARMD	11	+1.75/–1.50 × 5	+1.25/–1.75 × 10	1.00	0.82	3.8	Left
Trained	JH	89	Male	ARMD	7	+0.25/–1.00 × 100	+1.25/–0.75 × 75	0.78	0.68	1.2	Left
Trained	MS	75	Female	ARMD	6	+1.25/–0.75 × 75	–2.50/–0.75 × 75	1.06	0.60	0.7	Left
Trained	JG	75	Male	ARMD	6 mo	+1.25/–0.75 × 125	+1.75/–1.25 × 60	0.48	0.42	0.2	Left
Trained	DB	75	Female	ARMD	10	+2.50/–1.50 × 60	+1.75/–0.75 × 80	0.92	1.34	10.0	Right
Control	DS	69	Male	ARMD	5	+0.75/–1.00 × 65	–0.50/–0.50 × 110	0.60	1.64	1.0	Right
Control	SA^†^	88	Female	ARMD	5	+0.50/–1.50 × 80	pl/–1.00 × 100	0.40	0.68	1.2	Right
Control	AS	80	Female	ARMD	10	+0.50/–1.50 × 145	–0.75/–1.00 × 75	1.32	0.80	10.2	Left
Control	SS	69	Female	ARMD	8	+0.50/–2.50 × 90	–0.25/–2.00 × 80	0.20	0.20	0.7	Left
Control	SK	84	Male	ARMD	10	–0.25/–2.50 × 90	–1.00/–2.00 × 75	0.18	0.22	1.5	Right
Control	SG	72	Female	ARMD	14	+1.50/–2.75 × 85	+0.25/–1.75 × 90	0.88	0.84	9.0	Left
Control	GB	86	Male	ARMD	8	+1.50/–2.75 × 95	–1.25/–2.50 × 90	0.66	HM	0.9	Right
Control	RH	65	Female	JMD–Stargardt's disease	38	+1.00/–0.75 × 170	+0.25/–0.25 × 170	1.32	1.32	9.2	Right
Control	NT^‡^	59	Female	ARMD	22	–16.50/–1.25 × 95	–14.25/–2.00 × 130	0.62	NLP	0.9	Right

DS, diopter sphere (no astigmatism correction); NLP, no light perception;

pl, plano (no prescription needed); HM, hand movements; JMD, juvenile macular degeneration.

* The trained eye was determined by visual acuity and fixation stability measures.

† Participant who received an injection 1 day prior to post-training.

‡ Participant with ARMD in one eye and complete loss of vision in the other.

### Materials and Procedure: General

**Figure 2.**
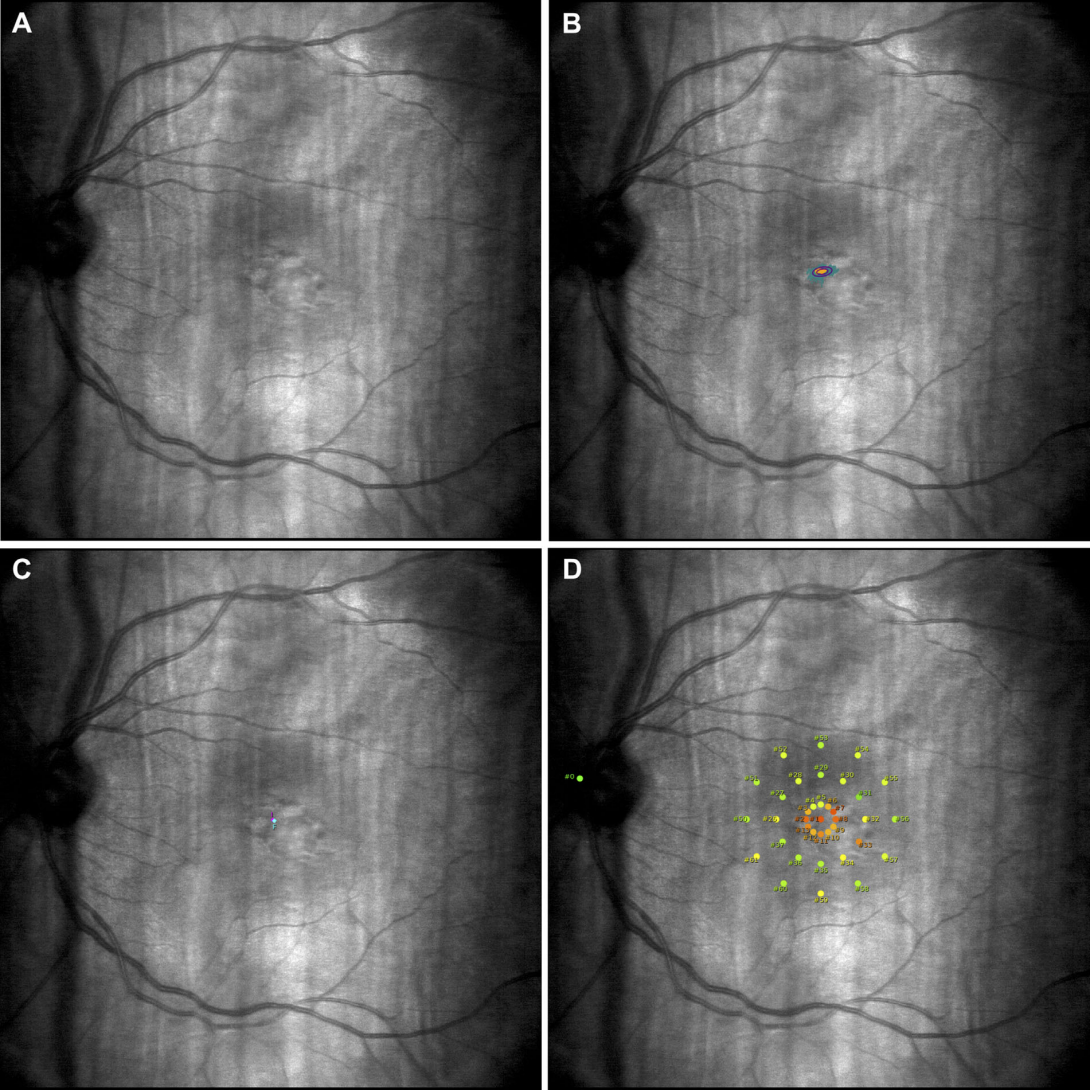
Left retina (**A**) and visual fixation area (**B**) of trained participant SC during microperimetry assessment. The cyan dots in **B** indicate fixation locations throughout the test, with the smaller *purple ellipse* signifying retinal stability for 63% of the test. (**C**) The participant's PRL; the *pink dot* illustrates the average retinal fixation locations during the first 10 seconds of the test, and the *cyan dot* illustrates the average PRL based on average fixations throughout the test. (**D**) The participant's retinal sensitivity map and the center of the optic nerve (the *green dot* on the left). The *orange dots* illustrate the least sensitive retinal areas, and the *green dots* illustrate the most sensitive regions.

Visual acuity was measured monocularly using Early Treatment Diabetic Retinopathy Study charts^45^ and was calculated in logMAR units.^46^ Fixation stability was measured monocularly and quantified using the bivariate contour ellipse area (BCEA); fixation stability, visual field sensitivity, and PRL were measured using a MAIA microperimetry device (CenterVue, Padova, Italy) (Fig. 2).^47^^,^^48^ If eyes had similar acuity, they were both tested on the MAIA, and the eye with greater fixation stability was then selected for training. The untrained eye was covered with a standard eye patch during training.

The experiment and stimuli were controlled and generated using PsychoPy^49^ and presented on a 40-inch Dell (Round Rock, TX, USA) Trinitron CRT P1130 monitor (resolution, 1280 × 1024 pixels; frame rate, 85 Hz; mean background luminance, 45 cd/m^2^, gamma-corrected; visual angle, 1.77′/pixel). A constant viewing distance of 60 cm was used and maintained by a forehead and chin rest. All subjects were optically corrected and provided with an appropriate near add for the viewing distance (+1.75 diopter sphere [DS]). Testing was performed in a darkened room.

Pre-training sessions lasted approximately 2 to 2.5 hours, training sessions approximately 1 hour, and post-training sessions approximately 1.5 to 2 hours; all included breaks. Pre- and post-training sessions for the trained group were 6 days apart, allowing 5 consecutive days of discrimination task training; for the control group, they were 6 days apart with no interim training.

### Materials and Procedure: Discrimination Task

**Figure 3.**
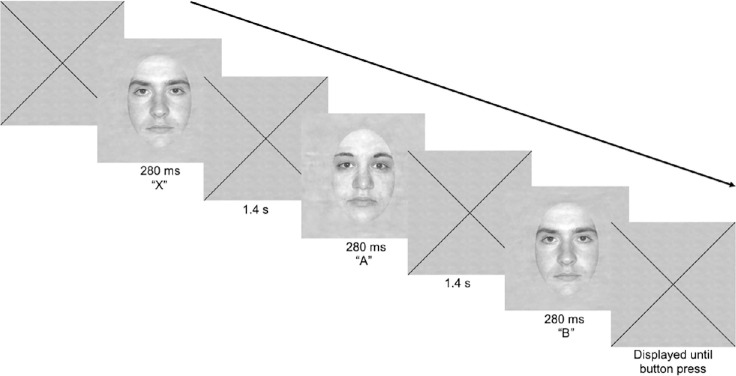
An example of a single XAB discrimination task trial. Participants fixated their PRL on the center of the fixation cross, present at the beginning of the trial and between stimuli. Participants were required to judge which of the last two images matched the first image they saw. When participants verbally expressed their choice (“A” or “B”), the experimenter pressed the corresponding button to elicit auditory feedback (correct responses, high-pitched tone; incorrect responses, low-pitched tone) and the next trial. The original face stimuli are available online at https://iuvislab.sitehost.iu.edu/IUVISIONLAB/publications.html.

This task was used as a pre- and post-training measurement and training task (Fig. 3). The methods to create the stimuli used in this study have been described in a previous paper.^50^ An additional set of stimuli with spatial characteristics similar to those of the original set of 10 faces was created for subsequent experiments.^51^^,^^52^ We used the full set of 20 grayscale oval faces used in Hussain et al.,^51^^,^^52^ including the static two-dimensional square Gaussian noise field (256 × 256 pixels) on which the faces were presented. These faces had neutral expressions (190:140 pixels), were cropped to display only internal features (10 male, 10 female), and, in the current study, were displayed from a maximum size of 37°.

Participants were asked to fixate on a central black cross and were then shown the task stimuli at sizes of 10°, 20°, 30°, and 37° to determine when they could see the stimuli (which indicated their starting point). They were then given a single practice run (3–4 minutes, ∼25 trials).

Discrimination performance was measured using an XAB design with a three-correct-down, one-incorrect-up adaptive staircase method converging on 79.4% accuracy. Trials began with a large black fixation cross and were initiated with a button press by the experimenter. Participants were asked to fixate on the center of the cross with their PRL as best they could (prior to each run), and fixation was continuously monitored by the experimenter, who was sitting to the side of the participant. Three faces were displayed sequentially, each for 280 ms, with an interstimulus interval of 1.4 seconds during which the fixation cross was again displayed. Participants had to decide whether face A or B (the last two faces) matched face X (the first face) and then verbally express their response (“A” or “B”) so the experimenter could press the corresponding button to elicit auditory feedback and the next trial. Correct responses elicited a high-pitched tone, incorrect responses a low-pitched tone. The task consisted of five runs of 50 trials (∼7 minutes), all undertaken in the same session. Accuracy was recorded using the size at which participants were able to correctly discriminate between faces. When correctly discriminated, faces were presented at a smaller size; incorrect discriminations resulted in faces being presented at a larger size.

### Materials and Procedure: Recognition Task

This task was used only in pre- and post-training sessions. Stimuli were adapted from a previously validated collection of 294 well-known faces from the past several decades,^18^^,^^53^ with an additional 169 country-specific faces added after separate pilot testing. Stimuli were grayscale on a gray background and sized at 20° when viewed from 60 cm—the distance at which most personal conversations take place.^54^ The horizontal width between pupils and vertical height between eyes and mouth was equivalent to ensure constant internal reference points across faces. Images with distinguishing external features were discarded.

Face recognition consisted of three phases. In phase 1 (pre-training), participants were required to correctly recognize 20 faces from a total of 463 faces using one of three familiarity ratings: familiar and able to be identified by name (e.g., “Margaret Thatcher”); familiar but unable to be named (i.e., identified by context, such as “She was the first female prime minister of the UK”); or unfamiliar (i.e., unable to be identified or identified incorrectly, such as “I have no idea who that is” or “She was an American singer”). When participants had identified 20 familiar faces by name, the task ended. Participants were encouraged to guess the names of faces that appeared familiar; a maximum of three guesses per image was permitted. If participants viewed all 463 faces and were unable to correctly recognize and identify 20 faces by name, faces that were recognized as familiar but unable to be named were used. If participants were still unable to correctly recognize 20 faces, only the faces they were able to recognize as familiar were used. No time or fixation restrictions were enforced; however, short breaks were encouraged.

Phase 2 (pre- and post-training) consisted of showing participants only the 20 faces from phase 1 that were recognized as familiar and identified by name (or recognized as familiar if unable to be named) in random order, with the experimenter explicitly naming each face. Phase 3 (pre- and post-training) consisted of showing participants the 20 faces from phase 2 in random order and asking participants to identify these faces by name. The images used in this phase differed from those in phase 2, as they were different versions of the faces that had previously been recognized as familiar and identified by name (e.g., a slightly younger/older image). The faces in this phase were originally presented at a height of 0.5°, and participants were required to adjust this size until the face was large enough to identify or to stop increasing the size if they were unable to recognize the face or if they recognized the face but were unable to name it. Again, three guesses were permitted. The images used in phase 3 for pre-training differed from images used for post-training to minimize memory effects.

### Statistical Analysis

For each of the five runs within a discrimination task training session, the last 6 reversals were used to calculate the face size threshold; the mean threshold of these five represented the daily performance threshold. Trained participants finished with seven estimates (pre- and post-training; 5 training days) and control participants with two estimates (pre- and post-training). These raw scores were then divided by participants’ pre-training performance score to produce a normalized score. A reduction in threshold size indicates improved discrimination performance. Repeated measures ANOVA (group × session) and Bonferroni-corrected multiple comparison Student's *t-*tests were used to assess the statistical significance of mean performance differences. Alpha values were set to 0.05, and all tests were two tailed.

## Results

Independent samples *t*-tests were conducted to determine if groups differed at baseline on age, acuity, fixation, and PRL variables; no significant differences between groups was found (age, *P* = 0.51, NS; acuity, *P* = 0.82, NS; fixation, *P* = 0.80, NS; PRL, *P* = 0.97, NS).

Pearson correlations were completed between visual measures at baseline and pre-training face tasks and change in performance from pre- to post-training to determine any associations among variables (Supplementary Table S1). As expected, PRL showed a moderate correlation with fixation stability (*r =* 0.50; *P* < 0.05) and visual acuity (*r =* 0.56; *P* < 0.01), and pre-training face recognition showed a moderate correlation with change in recognition performance (*r =* 0.49; *P* < 0.05) and a strong correlation with pre-training discrimination performance (*r =* 0.69; *P* < 0.01). Importantly, a moderate positive association was found between pre-training face performance and visual acuity (recognition: *r =* 0.50, *P* < 0.05; discrimination: *r =* 0.58, *P* < 0.01), and a moderate to strong association was found between pre-training face performance and PRL (recognition: *r =* 0.63, *P* < 0.01; discrimination: *r =* 0.62, *P* < 0.01). As such, these two variables were included as covariates into the discrimination and recognition analyses.

### Within and Between Task Learning

**Figure 4.**
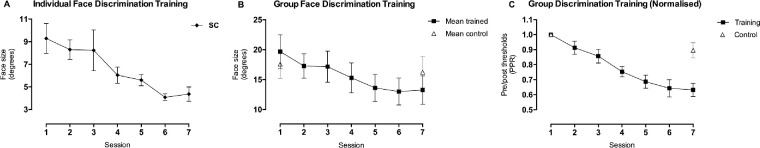
(**A**) An individual learning function for the discrimination task for participant SC, demonstrating typical improvement in performance across session and from pre- to post training. (**B**) Learning data illustrating the mean face size that was able to be discriminated by participants in each group over the training sessions. (**C**) Normalized pre- and post-training threshold learning data for each group. A decreasing face size/threshold indicates improved performance. Error bars represent ±1 SE.

Trained individuals showed reductions in threshold size at which faces were able to be discriminated across sessions, and a greater reduction in threshold size from pre- to post-training compared to controls (Table 2). Figure 4A illustrates the change in performance of a typical participant (SC) as training progressed. See Supplementary Figure S1 for individual participant learning curves. Figures 4B and 4C show raw and normalized threshold data for each group.

**Table 2.** Comparison of Group Performance Across Sessions^*^

	Training Group, Mean (SD)			Control Group, Mean (SD)		
Measurement	Pre-Training	Post-Training	Change in Performance^†^	Pre-Training	Post-Training	Change in Performance^†^
Discrimination task	19.7° (8.88°)	13.3° (7.47°)	–32.7%(–15.9)	17.0° (7.76°)	15.6° (8.54°)	–8.30% (10.1)
Recognition task	6.54° (1.85°)	5.08° (1.89°)	–22.4% (+2.31)	4.37° (2.68°)	4.47° (2.55°)	+2.36% (–5.12)
Fixation stability (BCEA 63%)	3.92°^2^ (4.70°^2^)	4.78°^2^ (6.85°^2^)	+0.22°^2^ (+0.46°^2^)	3.36°^2^ (3.93°^2^)	2.29°^2^ (2.40°^2^)	–0.32°^2^ (–0.39°^2^)
Visual acuity (logMAR)	0.66 (0.20)	0.66 (0.20)	0.00 (0.00)	0.63 (0.35)	0.68 (0.34)	+0.05 (–0.04)

* Figures refer to monocular data from the trained eye for the training group and from the eye with better fixation stability for the control group.

† For the two tasks, this was measured as a reduction in threshold size at which faces could be reliably discriminated or recognized.

To determine if perceptual training had any effect on the size at which a face could be discriminated, a 2 × 2 ANOVA was performed on the mean performance of individuals in pre- and post-training sessions (Fig. 4C). A significant interaction between group and session was found (*F*_1,15_ = 19.5; *P* *<* 0.001); there were no significant main effects. Further post hoc analysis showed that trained individuals showed a significant reduction in threshold size from pre- to post-training (*t*_9_ = 8.27; *P* *<* 0.001), whereas the control group showed no such reduction in threshold size (*t*_8_ = 1.92; *P* = 0.09, NS), indicating that perceptual training reduced the size at which faces can be reliably discriminated in patients with ARMD.

**Figure 5.**
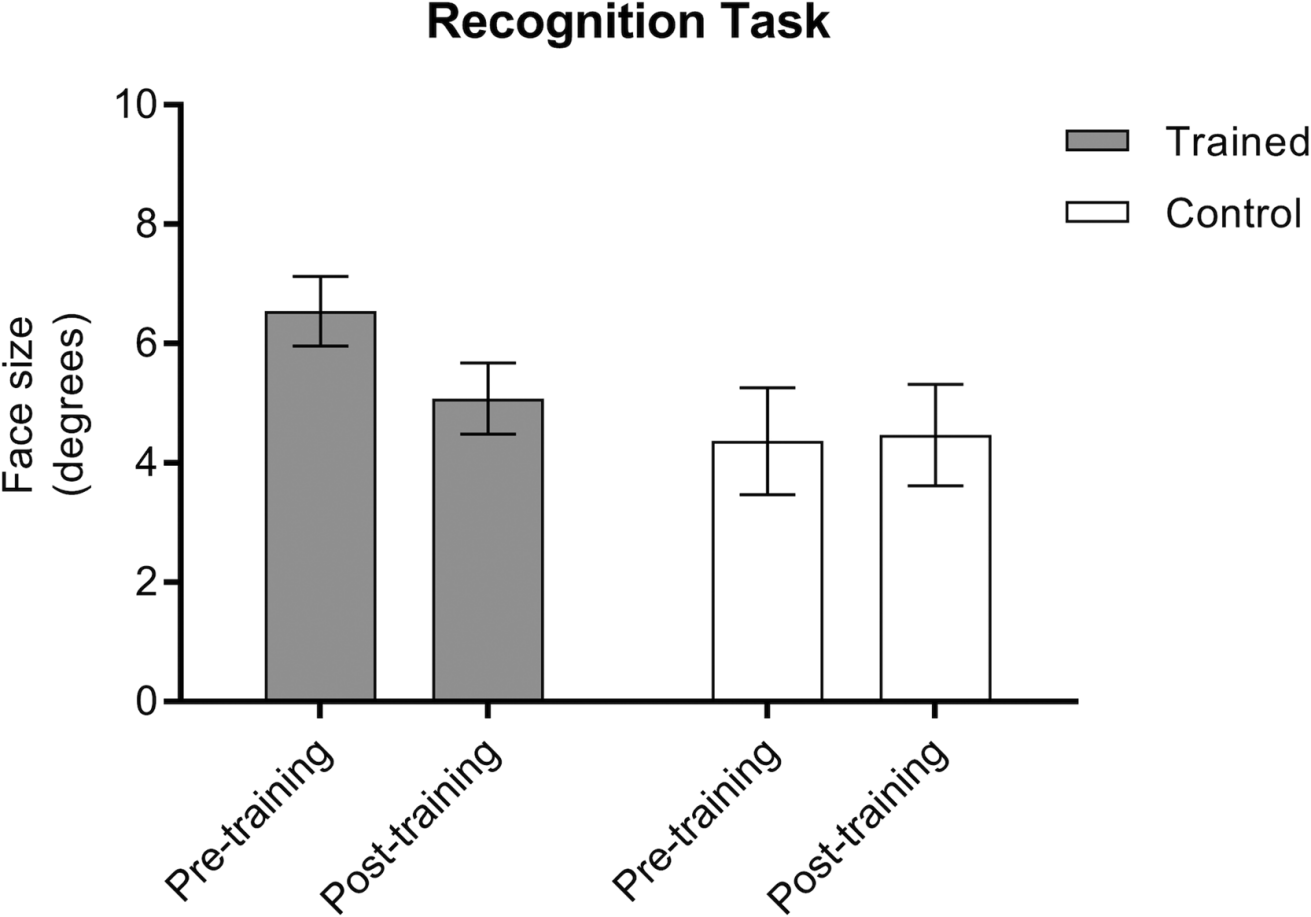
Average size at which participants were able to recognize famous faces with which they were already familiar, shown for both groups in pre- and post-training sessions. Error bars represent ±1 SE.

To establish the transfer of training-based reductions in threshold size for face discrimination, participants also performed a face recognition task. Of the 19 participants, three were unable to recognize as familiar a total of 20 faces required for the task in phase 1, either named or unnamed faces: AS-1 = 17 and DB = 17 in the trained group, and RH = 8 in the control group; however, they were still included in this analysis. Only faces that were able to be recognized as familiar (i.e., identified by name or context) within both pre- and post-training sessions were included in these analyses; for example, if Margaret Thatcher was recognized in phase 3 of pre-training but not in phase 3 of post-training, then her image was removed from the analysis for that participant. The number of faces recognized in phase 3 did not show an interaction between group and session from pre- to post-training (*F*_1,17_ = 0.12; *P* = 0.74, NS), nor was there any difference in the number of faces able to be recognized in phase 3 from pre- to post-training between groups (*F*_1,17_ = 0.90; *P* = 0.36, NS) or within groups (*F*_1,17_ = 0.18; *P* = 0.68, NS). On average, the training group correctly recognized and identified 90% of faces in phase 3, whereas the control group correctly recognized and identified 93% of faces. A reduction in threshold size necessary to support successful face recognition in the post-training session was also demonstrated in the trained group, but not in the control group (Table 2). A 2 × 2 ANOVA on the mean performance of individuals in pre- and post-training sessions (Fig. 5) revealed a significant interaction between group and session (*F*_1,15_ = 8.25; *P* = 0.012) and a significant between-subjects main effect of PRL (*F*_1,15_ = 4.55; *P* = 0.05). Post hoc analyses demonstrated a significant decrease of 22% in the size needed for face recognition in the post-training session in the trained group (*t*_9_ = 3.44; *P* = 0.007) but a small non-significant size increase of 2% in the control group (*t*_8_ = 0.23; *P* = 0.83, NS). Parameter estimates for the main effect demonstrate that, although PRL was associated with face recognition performance at pre-training (β = 0.24; *P* = 0.02), this was not the case at post-training (β = 0.11; *P* = 0.24, NS).

These results demonstrate that the eccentricity of the PRL at pre-training was 0.25° greater for every 1° increase in the size of the face being recognized for both groups, but this was not the case at post-training. Furthermore, it suggests that training-induced reductions in threshold size for face discrimination show considerable transfer to face recognition and can facilitate identification of faces on a smaller spatial scale or at an increased distance.

### Fixation Stability

**Figure 6.**
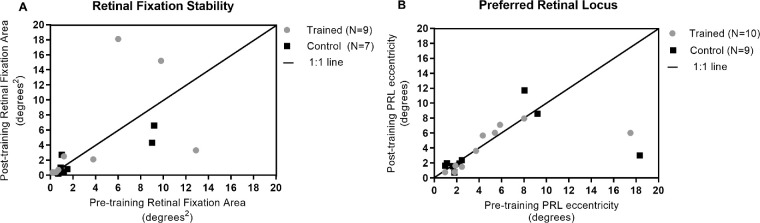
(**A**) Pre- and post-training average fixation areas for each participant measured using the MAIA. Three participants were excluded from this analysis due to problems imaging their optic disc. Most participants showed no change from pre- to post-training; those who did showed no systematic pattern (trained: MV, WB, RT), indicating some individual variability. (**B**) Pre- and post-training average eccentricity of the PRL for each participant relative to the anatomical fovea. Two participants (trained: DB; control: AS) showed a relatively large shift in PRL. The 1:1 line is plotted in both figures. Retinal maps for individual participants’ fixation stability and PRL are provided in Supplementary Figure S2.

To check whether reductions in threshold size for face discrimination were due to changes in fixation patterns, fixation stability was measured pre- and post-training using the MAIA. Due to technical difficulties in imaging the optic nerve head, one participant from the trained group (DB) and two from the control group (AS and NT) were excluded from these analyses. The average fixation area showed little or no change from pre- to post-training in either group (Table 2). A 2 × 2 ANOVA on mean fixation stability showed no evidence of an interaction between group and session (*F*_1,14_ = 0.70; *P* = 0.42, NS) and no significant change in the distribution of fixation from pre- to post-training within groups (*F*_1,14_ = 0.009; *P* = 0.93, NS) nor between groups (*F*_1,14_ = 0.48; *P* = 0.50, NS) (Fig. 6A).

### Preferred Retinal Locus

To determine whether training-based reductions in threshold size were due to participants adopting a new or improved retinal locus for viewing, changes in the eccentricity of the PRL were measured and calculated using the average PRL location and *x*,*y* coordinates relative to the anatomical fovea (as identified in all participants from the MAIA) (Fig. 6B). The average PRL eccentricity showed no change in the trained group from pre-training (mean = 5.19°; SD = 4.84°) to post-training (mean = 4.11°; SD = 2.77°) nor in the control group (pre-training: mean = 5.09°, *SD* = 5.83°; post-training: mean = 3.73°, SD = 3.78°). A 2 × 2 ANOVA on mean PRL eccentricity showed that the average PRL eccentricity of all participants demonstrated no change from pre-to post-training (*F*_1,17_ = 1.34; *P* = 0.26, NS) or between groups (*F*_1,17_ = 0.02; *P* = 0.89, NS). The average distance of the PRL from the anatomical fovea also showed no change for the trained group (pre-training: mean = 14.8°, *SD* = 3.73°; post-training: mean = 14.4°, SD = 3.41°) or the control group (pre-training: mean = 15.4°, SD = 4.24°; post-training: mean = 15.0°, SD = 2.82°). This indicates that reductions in threshold size on the face tasks in the trained group cannot be explained by the adoption of a different, or more preferable, retinal location. See Supplementary Figure S2 for individual participant MAIA images referencing fixation and PRL locations.

### Visual Acuity

**Figure 7.**
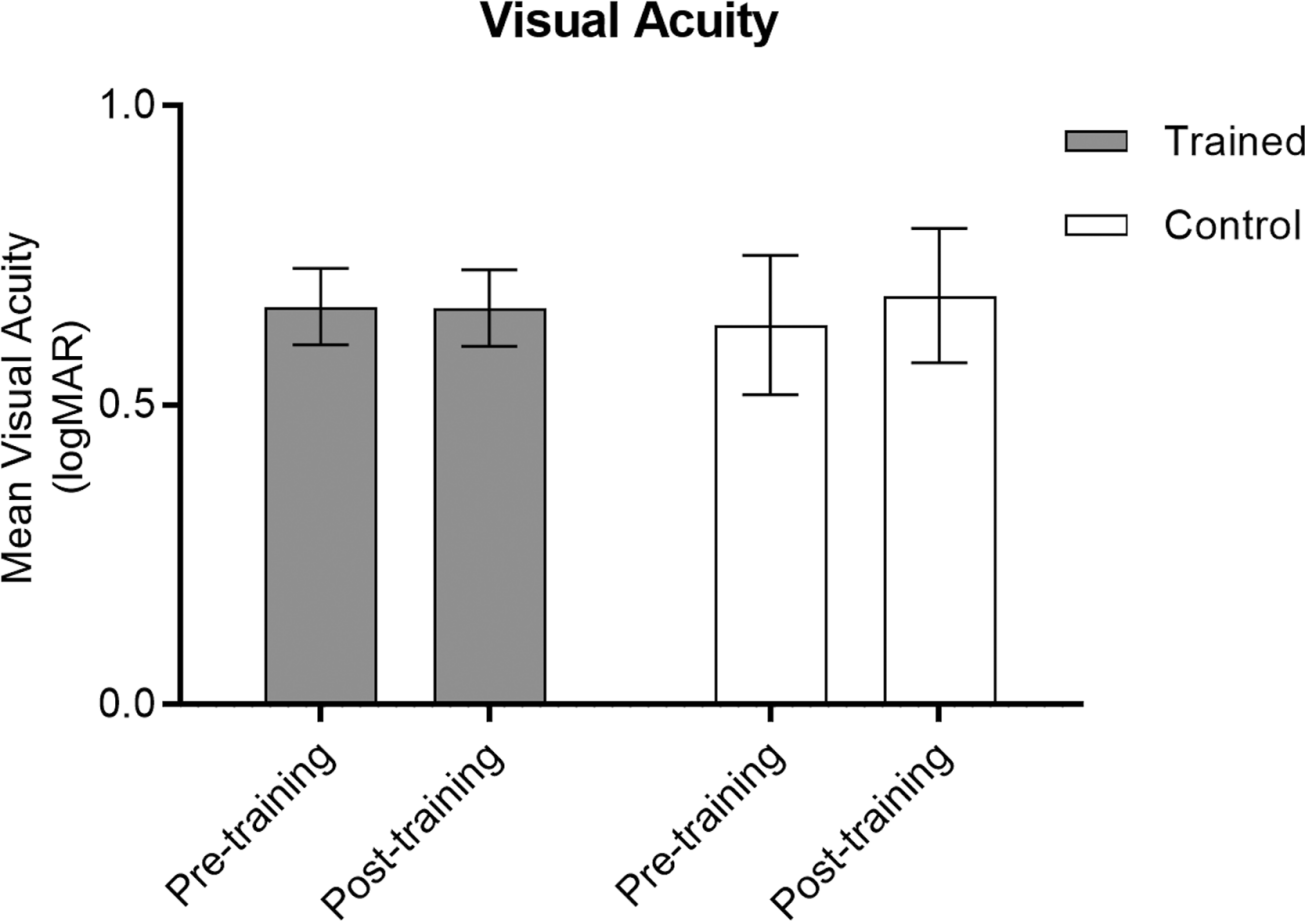
Average logMAR visual acuity measured at pre- and post-training sessions. Acuity estimates were stable for both training and control groups. Error bars represent ±1 SE.

Finally, to investigate whether changes in visual acuity underlie improvements on the face tasks, visual acuity was also measured pre- and post-training. No change in mean visual acuity was evident for either group (Table 2), with a 2 × 2 ANOVA confirming no significant change from pre- to post-training (*F*_1,17_ = 0.81; *P* = 0.38, NS) (Fig. 7) or between groups (*F*_1,17_ = 0.001; *P* = 0.97, NS). Therefore, reductions in threshold size for face discrimination and recognition appear independent of changes in visual acuity.

## Discussion

This study demonstrates that perceptual training on a face discrimination task reduces threshold size for face discrimination in individuals with established ARMD. Furthermore, this improvement generalized to a famous-face recognition task—reducing the size at which faces could be reliably recognized—but not to measures of visual acuity. We found no changes in fixation stability or PRL, suggesting it is unlikely these factors contributed to the reliable reduction in threshold size on both face-based tasks. The control group, who did not train on the face discrimination task, showed no reductions in threshold size for the face discrimination task nor any transfer to the face recognition task, and their fixation patterns also remained largely unchanged. In the vast majority of individuals with central vision loss (∼84%), adaptation to macular damage involves developing a surrogate fovea or PRL,^12^^,^^55^ which supports better fixation^56^ and improved reading task performance.^11^ We have shown for the first time, to the best of our knowledge, that perceptual learning can be used to also improve face discrimination and recognition in individuals with ARMD.

It was previously thought that words were identified by parts and faces as wholes,^57^ but recent evidence suggests that both are similarly processed by parts and suffer from common functional constraints, such as internal crowding and visual span.^58^^,^^59^ It is well known that crowding increases significantly with retinal eccentricity and becomes a major bottleneck to object identification in the periphery.^60^ The current study lends support to this line of reasoning for face recognition, as the positive association between pre-training face recognition performance and PRL in the total sample explained some of the variation in baseline face recognition, indicating worse recognition with increasing eccentricity. When viewed in the periphery or with a PRL, the internal features of a face may not be sufficiently separated to avoid crowding, thus contributing to impaired face recognition in ARMD. However, because the association between face recognition performance and PRL was not significant at post-training, it is possible that the improvements across both tasks arose from an alleviation of this internal crowding and were not due to the adoption of an improved PRL.

Work in the visual periphery has shown that crowding effects can be reduced via perceptual learning.^61^ However, quantitative changes in the amount of crowding experienced in the peripheral field require specific training using continuous reductions in inter-element separation.^61^ The training objective of our study was to reduce the overall size required to support reliable face discrimination; by reducing size, the spacing between internal facial features would also contract. If these elements are being used to support discrimination, then the same perceptual learning mechanisms might operate. However, although a reduction of crowding effects may be the mechanism driving greater performance, this is confounded by the fact that the spacing and size of internal facial features are altered proportionally to each another. To clarify this issue, further research could benefit from training crowding for facial features by changing the spacing of features but holding their size constant.

Due to the behavioral and social importance of faces, humans need to retain the ability to recognize and discriminate new faces across the lifespan. Although the ability to encode faces first develops during a critical period early in life,^62^ two notable studies have demonstrated the plasticity of neural mechanisms involved in face recognition in later years and the essential role of experience in face recognition. Ostrovsky and colleagues^63^ measured the face discrimination of a woman who was blind for the first 12 years of life. After sight restoration, her visual acuity remained largely unchanged. Although not able to recognize people immediately, after 6 months she was able to recognize her siblings and parents, and on a face perception task she performed with high reliability despite visual factors such as illumination impacting her performance. A more recent study by Gandhi and colleagues^64^ measured the visual ability of congenitally blind children who regained sight as teenagers. After sight restoration, their visual acuity also remained compromised, as did their ability to discriminate between faces and non-faces. When participants returned several times for face discrimination testing, they showed graded responses to face stimuli over the course of 6 months, which led to a marked improvement in the categorical discrimination of faces.

The results from these studies draw parallels with our own in illustrating that visual experience appears to play a significant role in the improvement of face perception. For our participants, it appears that targeted visual experience—directing participants to focus on the internal features of a face—plays an important role in the functional recovery of face discrimination and recognition despite visual deprivation of this skill in the ARMD population. Collectively, these results suggest that there may be neural pathways involved in face processing that remain plastic throughout life but require nurturing through experience in order to function. Such pathways may be able to be exploited through different learning-based strategies to develop face recognition ability later in life or when previous strategies are no longer useful.

In discussing the results of this study, a few points must be kept in mind. The first point concerns the fact that fixation was monitored by the experimenter and not by any eye-tracking equipment. Despite there being no changes to the fixation patterns for trained participants, that there were reductions in threshold size for both face tasks from pre- to post-training could suggest two things—either that the improvement was not due to the adoption of a change in fixation pattern throughout training or that fixation patterns for face discrimination and recognition differ from fixation patterns recorded using the MAIA. As the adoption of task-dependent PRLs^65^^,^^66^ could account for improved performance on the face tasks, future studies would benefit from quantitatively measuring fixation throughout the task to determine the exact fixation pattern of participants.^14^ The second limitation is that training was monocular while real-world viewing is binocular. Although training may have helped participants to adopt a better monocular strategy, this may not transfer to binocular recognition of faces. Yet, a study by Kambanarou^67^ in ARMD patients found no difference in eye movements during monocular and binocular reading, even though some participants used different PRLs, and the monocular reading speed of the stronger eye predicted the binocular reading speed. Future studies would benefit from incorporating both monocular and binocular training and assessment measures which would allow even further investigation of the perceptual strategies underlying face recognition in ARMD.

### Summary

Perceptual learning is currently used for training individuals with ARMD on reading tasks,^11^^,^^68^ but it has not been used to improve face recognition. As this is a key symptom identified by ARMD patients^69^ and an essential aspect of maintaining social relations and quality of life,^3^ it is important to find ways to improve this skill. Here, we have shown that training-based reductions in the threshold at which faces can be reliably discriminated and recognized in ARMD patients are independent of visual acuity and oculomotor control. At present, it is not clear whether these benefits arise from reductions in internal crowding or by latent plasticity of face-processing neural networks. It is essential to establish this in order to optimize training protocols and further improve rehabilitation potential.

## Supplementary Material

Supplement 1

Supplement 2

Supplement 3
