# Supplementary material for: The Effect of Perceptual Learning on Face Recognition in Individuals with Central Vision Loss
Source: Invest Ophthalmol Vis Sci. 2020 Jul 1;61(8):2. doi: 10.1167/iovs.61.8.2 (PMC7425703; doi:10.1167/iovs.61.8.2)
Supplement: Supplement 1 [file iovs-61-8-2_s001.pdf]

Supplementary Table ST1. Correlations between vision variables and pre-training performance and improvement in performance on the face tasks.†

Measurement	1	2	3	4	5	6	7	8
Age	1.00	-	-	-	-	-	-	-
Fixation Stability (BCEA 63%)	-0.42	1.00	-	-	-	-	-	-
Visual Acuity (logMAR)	-0.29	0.25	1.00	-	-	-	-	-
Preferred Retinal Locus	-0.11	0.50*	0.56**	1.00	-	-	-	-
Pre-training face recognition performance	0.07	0.37	0.50*	0.63**	1.00	-	-	-
Change in face recognition performance	-0.03	0.17	-0.17	0.20	0.49*	1.00	-	-
Pre-training face discrimination performance	0.31	0.04	0.58**	0.62**	0.69**	0.10	1.00	-
Change in face discrimination performance	0.02	-0.10	-0.14	0.00	-0.33	-0.41	-0.32	1.00

† Pearson correlations were performed.
BCEA 63% (Bivariate Contour Ellipse Area, 63%).
logMAR (Logarithm of the Minimum Angle of Resolution).
** $p<0.01$; * $p<0.05$.
