# Supplementary material for: The Effect of Perceptual Learning on Face Recognition in Individuals with Central Vision Loss
Source: Invest Ophthalmol Vis Sci. 2020 Jul 1;61(8):2. doi: 10.1167/iovs.61.8.2 (PMC7425703; doi:10.1167/iovs.61.8.2)
Supplement: Supplement 2 [file iovs-61-8-2_s002.pdf]

Supplementary Figure SF1. Individual face discrimination training curves.

Trained Participants

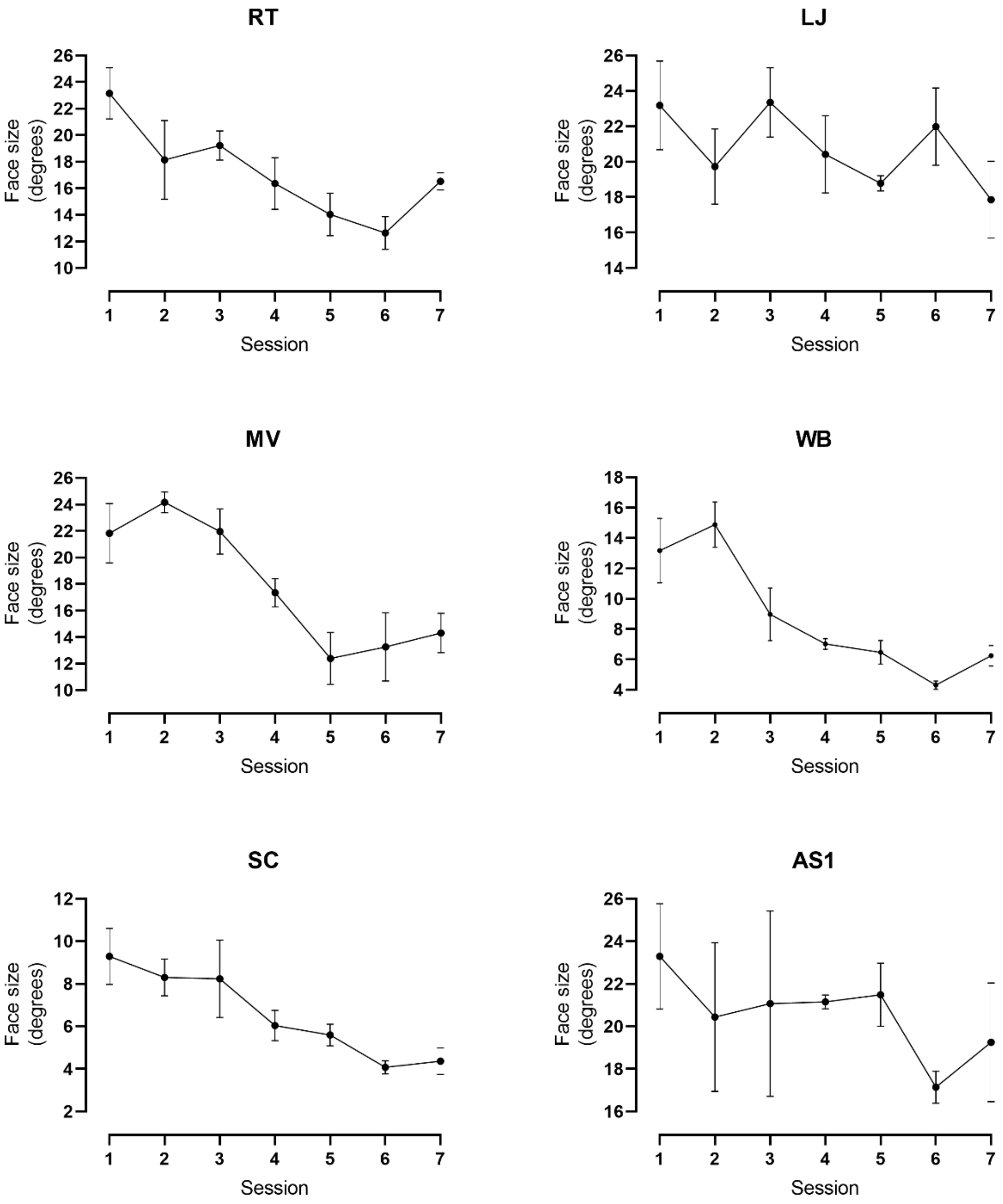

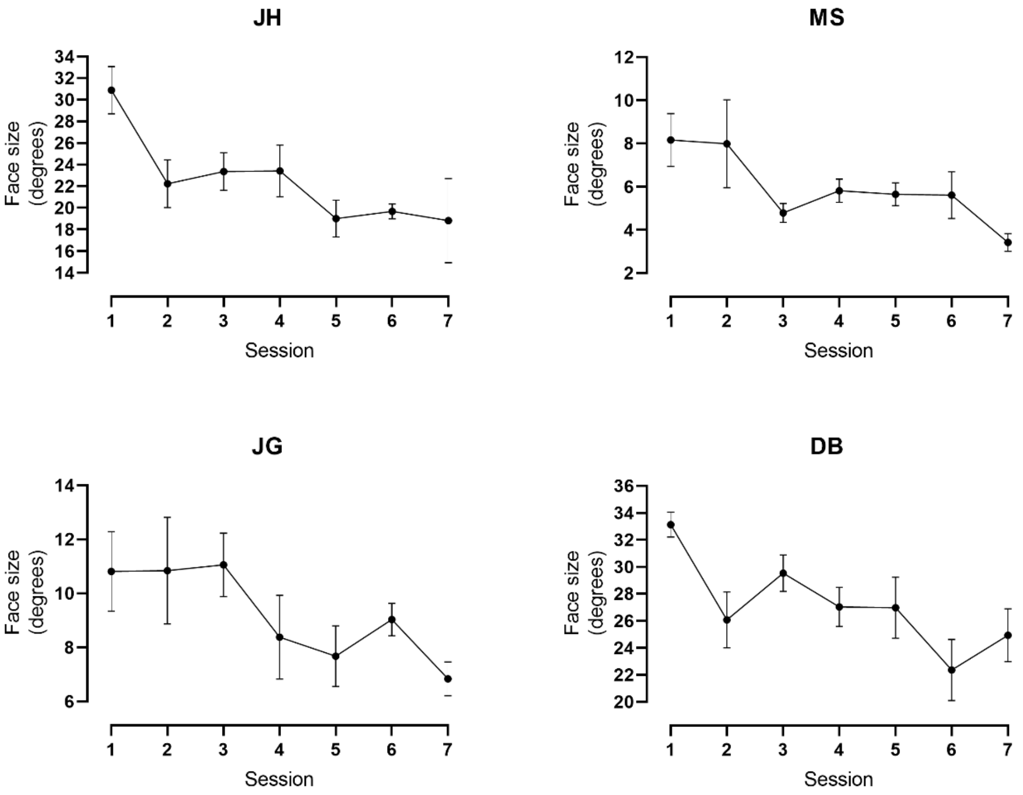

Control Participants

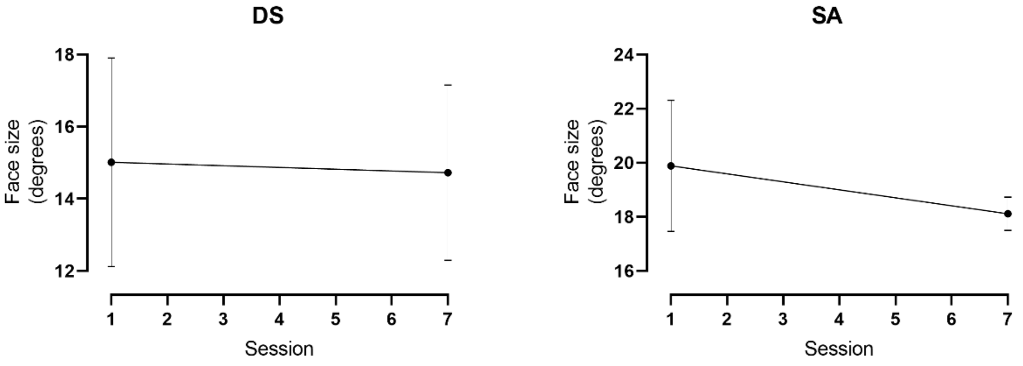

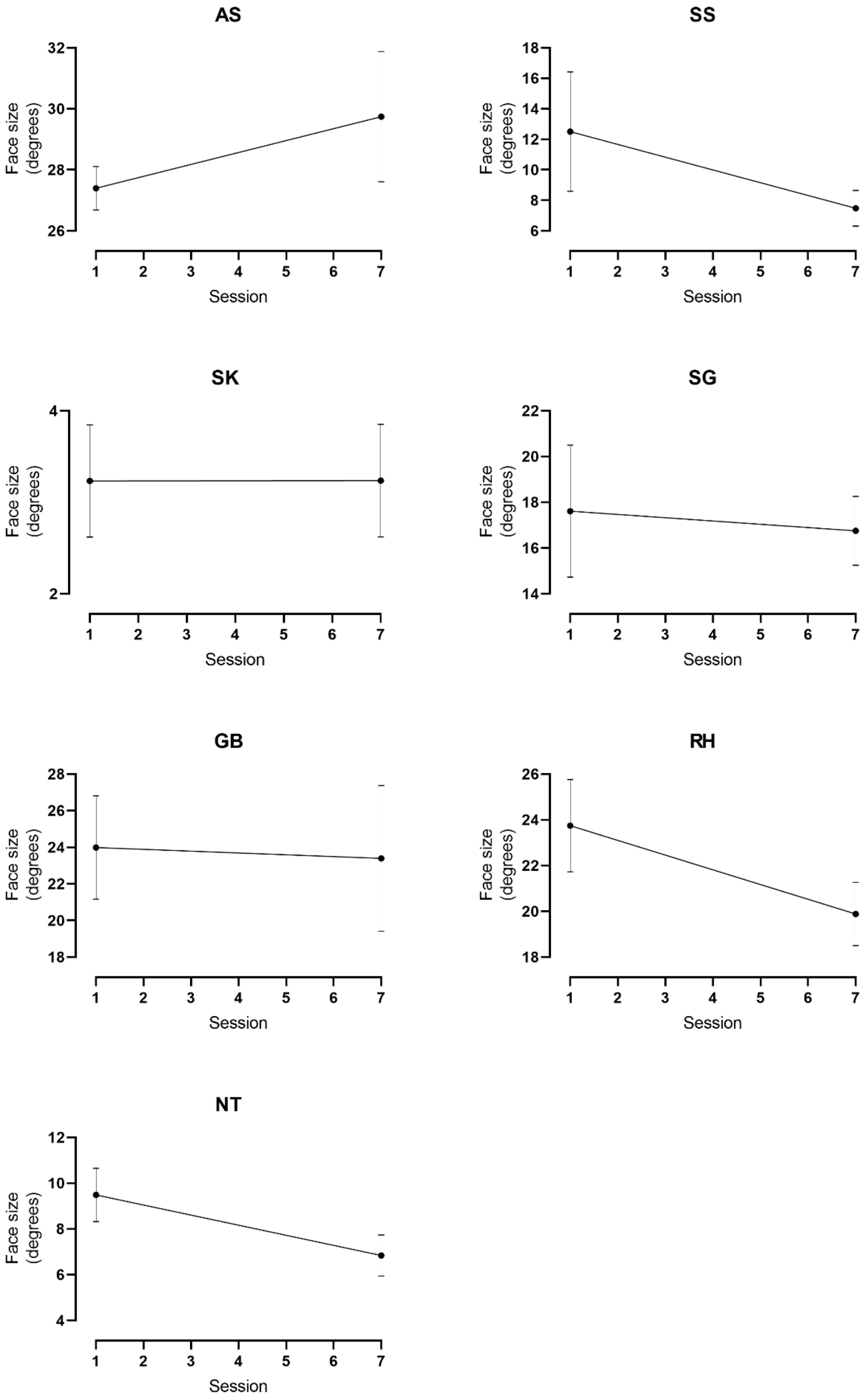

AS
Face size (degrees)
32
30
28
26
1 2 3 4 5 6 7
Session
SS
Face size (degrees)
18
16
14
12
10
8
6
1 2 3 4 5 6 7
Session
SK
Face size (degrees)
4
2
1 2 3 4 5 6 7
Session
SG
Face size (degrees)
22
20
18
16
14
1 2 3 4 5 6 7
Session
GB
Face size (degrees)
28
26
24
22
20
18
1 2 3 4 5 6 7
Session
RH
Face size (degrees)
26
24
22
20
18
1 2 3 4 5 6 7
Session
NT
Face size (degrees)
12
10
8
6
4
1 2 3 4 5 6 7
Session
