# Supplementary material for: The Effect of Perceptual Learning on Face Recognition in Individuals with Central Vision Loss
Source: Invest Ophthalmol Vis Sci. 2020 Jul 1;61(8):2. doi: 10.1167/iovs.61.8.2 (PMC7425703; doi:10.1167/iovs.61.8.2)

Supplementary Figure SF2. Fundus images from the MAIA showing the PRL (both left images) and fixation areas (both right images) for the pre-training session (first and third images) and post-training session (second and fourth images) for each individual participant.

* I (purple dot) = preferred retinal locus (PRL) for the first 10s of the microperimetry test.

* F (cyan dot) = average PRL for the complete microperimetry test.

* Small magenta circle = area of stable fixation for 63% of the microperimetry test.

* Large magenta circle = area of stable fixation for 95% of the microperimetry test.

Trained Participants

RT

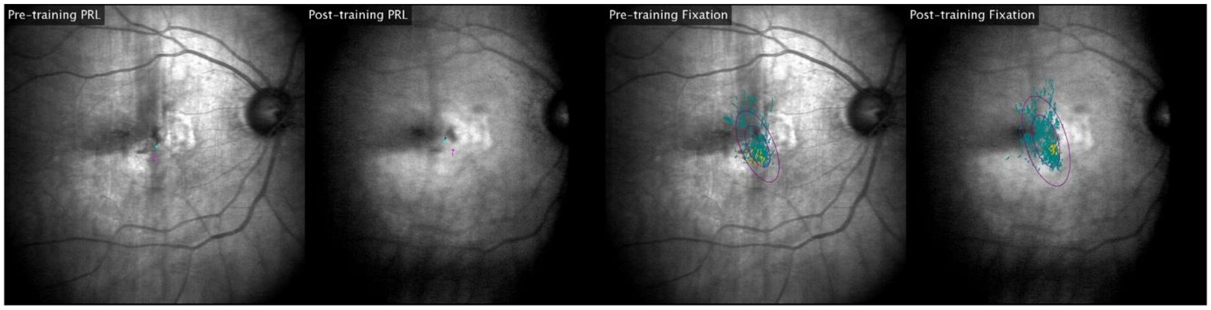

LJ

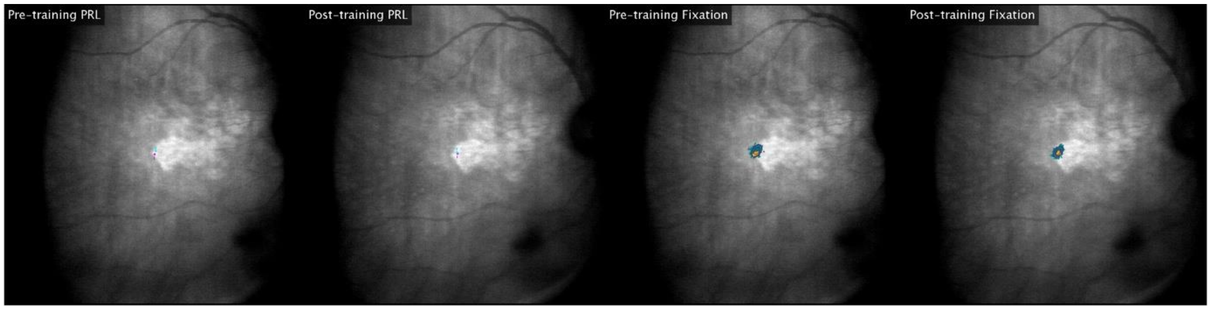

MV

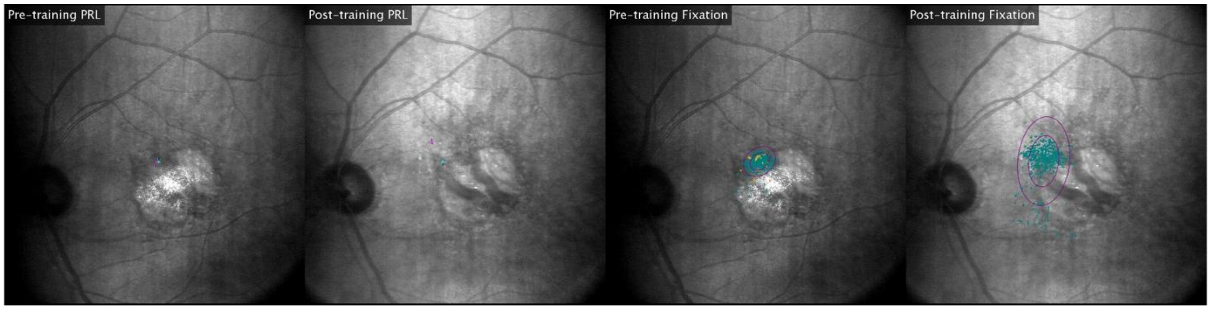

Pre-training PRL
Post-training PRL
Pre-training Fixation
Post-training Fixation

WB

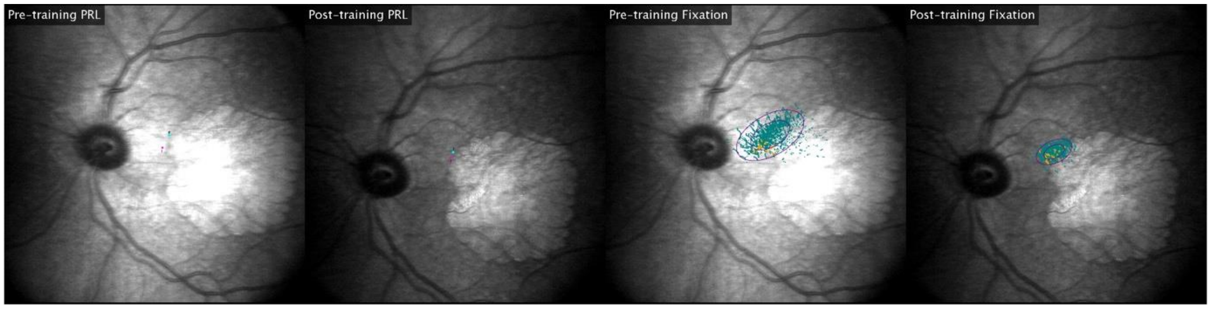

Pre-training PRL
Post-training PRL
Pre-training Fixation
Post-training Fixation

SC

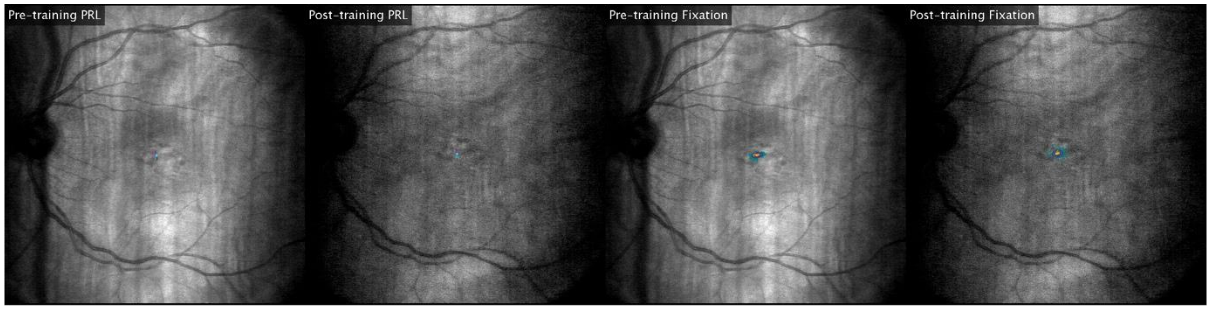

Pre-training PRL
Post-training PRL
Pre-training Fixation
Post-training Fixation

AS1

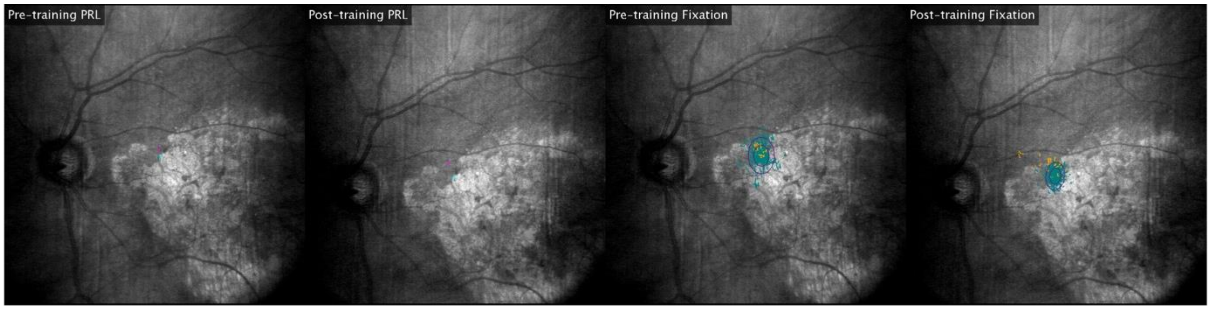

Pre-training PRL
Post-training PRL
Pre-training Fixation
Post-training Fixation

JH

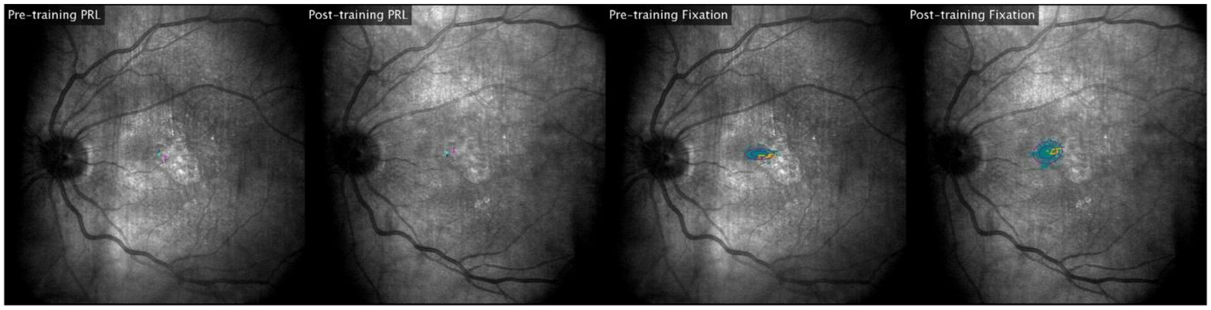

MS

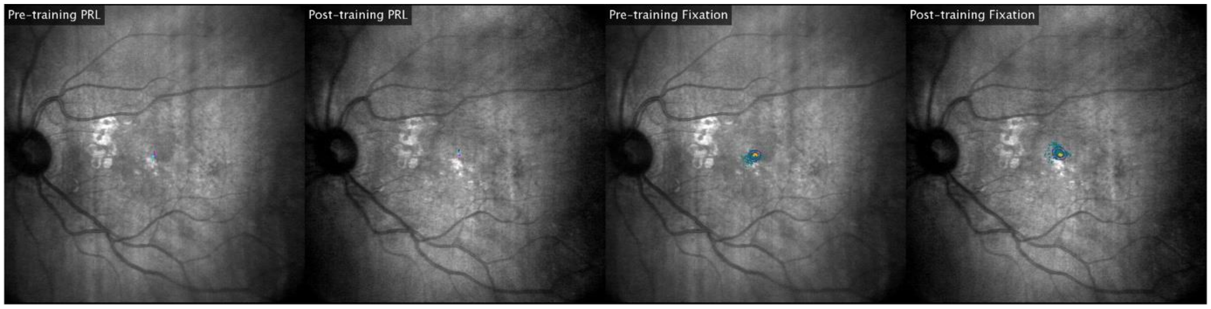

JG

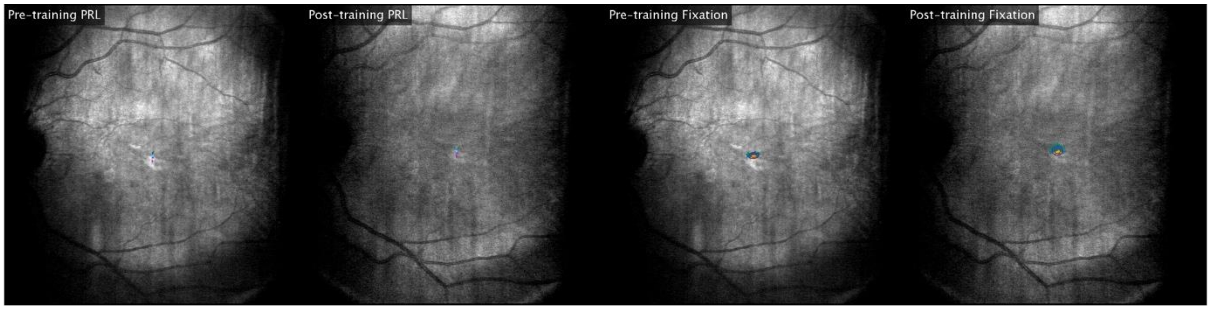

DB

* Optic disc unable to be imaged. Fixation stability not included in analysis.

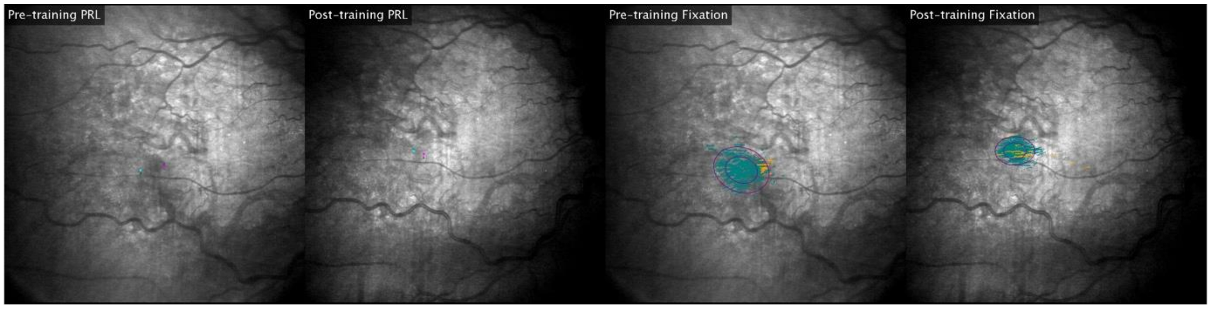

Control Participants

DS

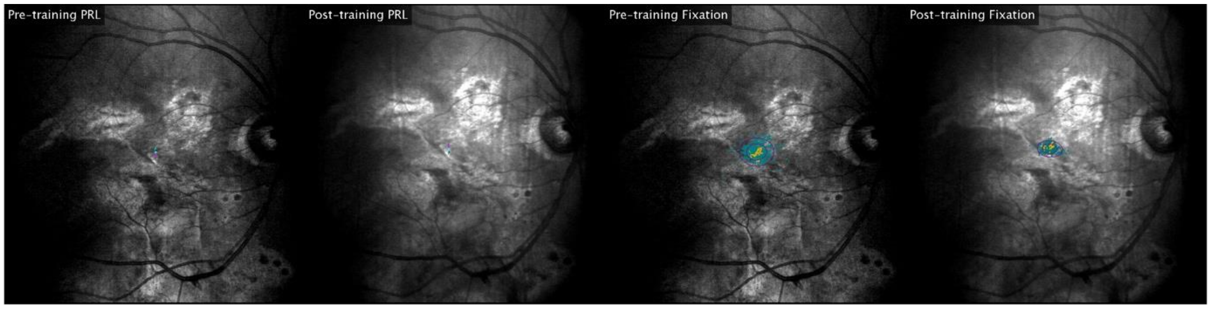

SA

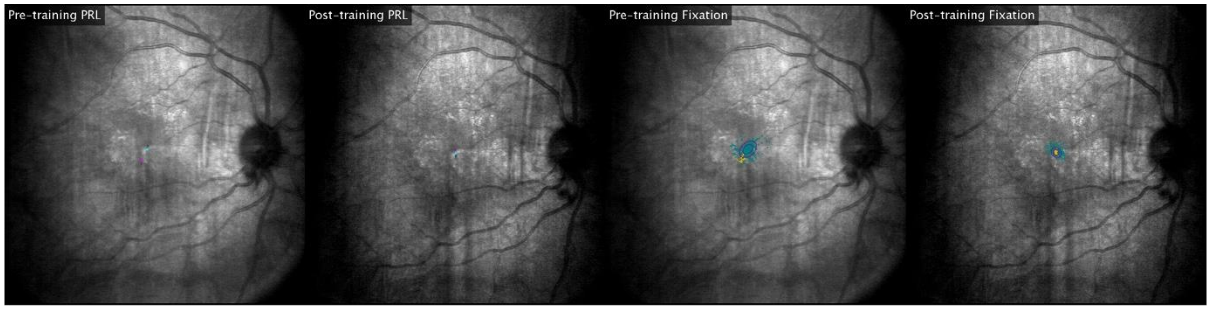

AS

* Optic disc unable to be imaged. Fixation stability not included in analysis.

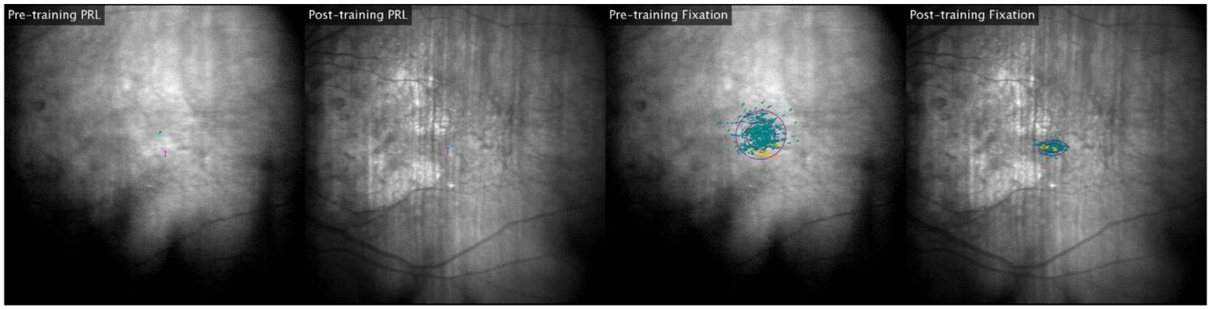

SS

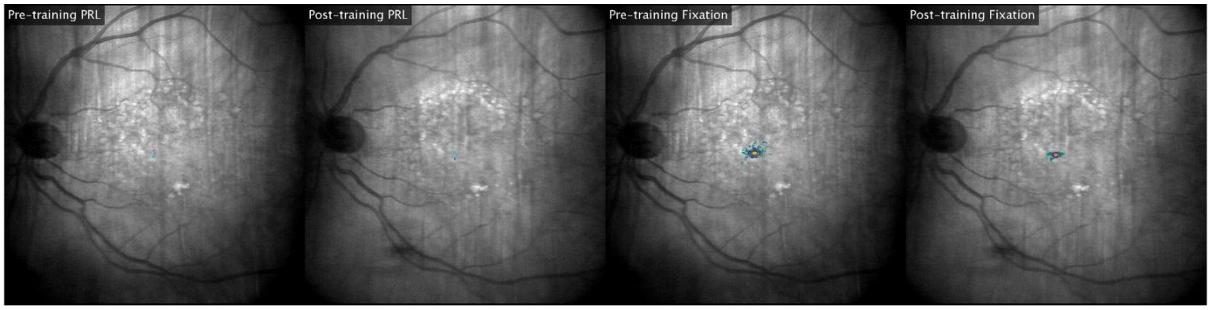

SK

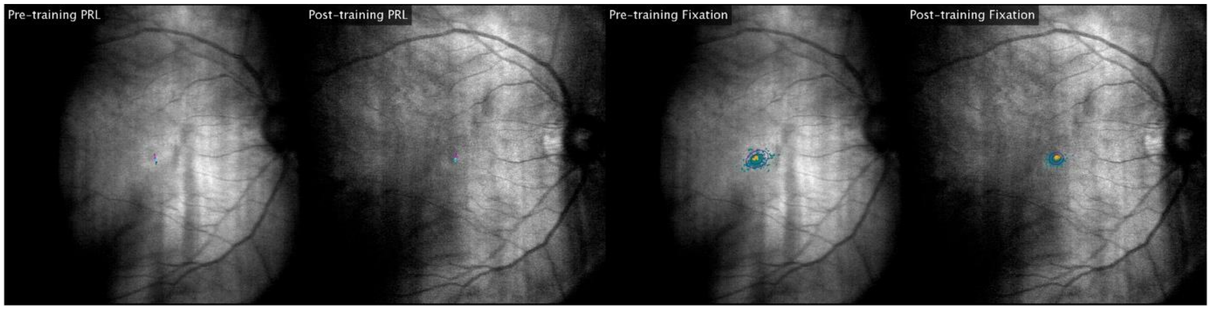
Pre-training PRL
Post-training PRL
Pre-training Fixation
Post-training Fixation

SG

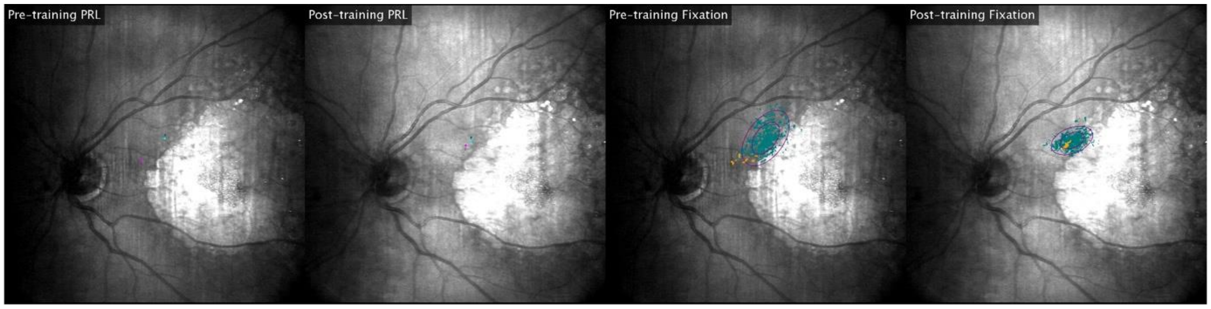
Pre-training PRL
Post-training PRL
Pre-training Fixation
Post-training Fixation

GB

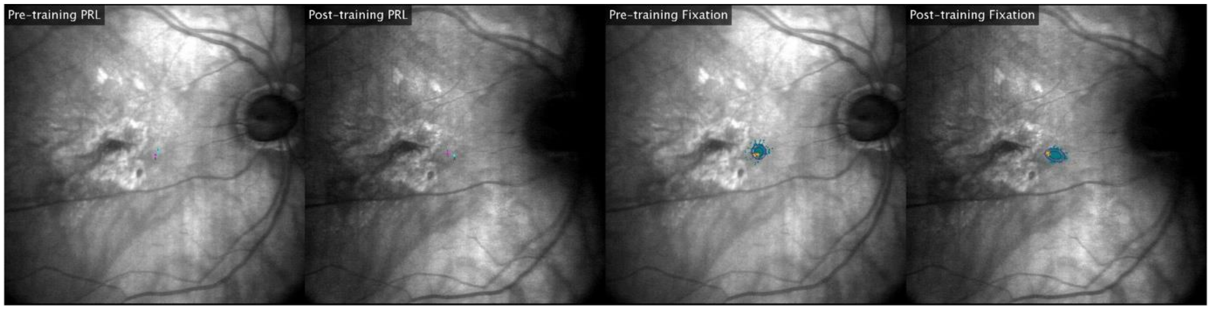
Pre-training PRL
Post-training PRL
Pre-training Fixation
Post-training Fixation

RH

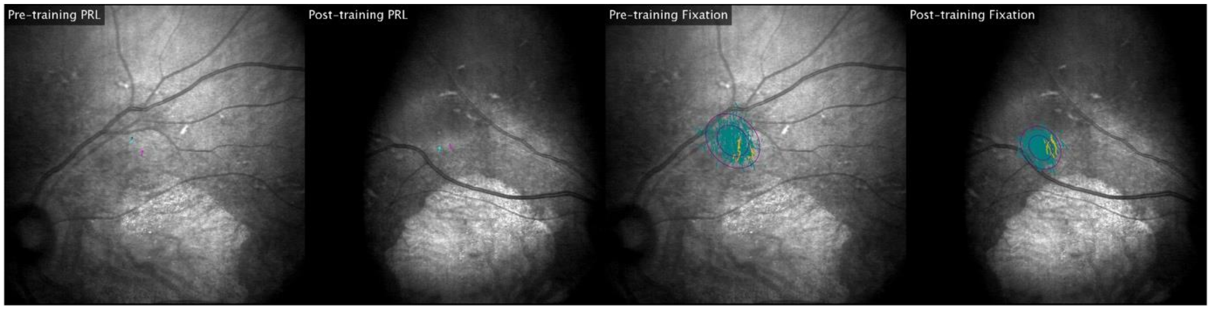
Pre-training PRL
Post-training PRL
Pre-training Fixation
Post-training Fixation

NT

* Optic disc unable to be imaged. Fixation stability not included in analysis.

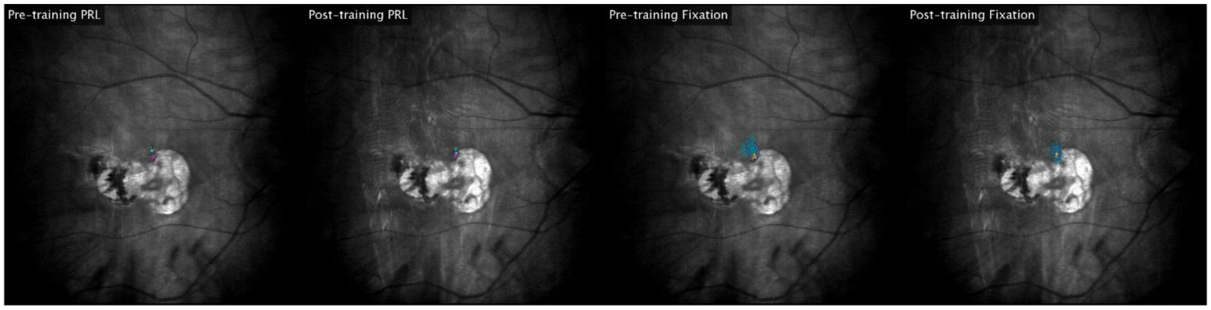

Supplement: Supplement 3 [file iovs-61-8-2_s003.pdf]
